# Increased amyloid β-peptide uptake in skeletal muscle is induced by hyposialylation and may account for apoptosis in GNE myopathy

**DOI:** 10.18632/oncotarget.7997

**Published:** 2016-03-08

**Authors:** Mònica Bosch-Morató, Cinta Iriondo, Biuse Guivernau, Victòria Valls-Comamala, Noemí Vidal, Montse Olivé, Henry Querfurth, Francisco J. Muñoz

**Affiliations:** ^1^ Laboratory of Molecular Physiology, Department of Experimental and Health Sciences, Universitat Pompeu Fabra, Barcelona, Spain; ^2^ Institut de Neuropatologia, Servei Anatomia Patològica, Hospital de Bellvitge, Hospitalet de Llobregat, Barcelona, Spain; ^3^ Department of Neurology, Rhode Island Hospital, Warren Alpert Medical School of Brown University, Providence, RI, USA

**Keywords:** Aβ, Akt, apoptosis, endocytosis, GNE myopathy, Gerotarget

## Abstract

GNE myopathy is an autosomal recessive muscular disorder of young adults characterized by progressive skeletal muscle weakness and wasting. It is caused by a mutation in the UDP-N-acetylglucosamine 2-epimerase/N-acetylmannosamine kinase (*GNE*) gene, which encodes a key enzyme in sialic acid biosynthesis. The mutated hypofunctional GNE is associated with intracellular accumulation of amyloid β-peptide (Aβ) in patient muscles through as yet unknown mechanisms. We found here for the first time that an experimental reduction in sialic acid favors Aβ_1-42_ endocytosis in C2C12 myotubes, which is dependent on clathrin and heparan sulfate proteoglycan. Accordingly, Aβ_1-42_ internalization in myoblasts from a GNE myopathy patient was enhanced. Next, we investigated signal changes triggered by Aβ_1-42_ that may underlie toxicity. We observed that p-Akt levels are reduced in step with an increase in apoptotic markers in GNE myopathy myoblasts compared to control myoblasts. The same results were experimentally obtained when Aβ_1-42_ was overexpressed in myotubes. Hence, we propose a novel disease mechanism whereby hyposialylation favors Aβ_1-42_ internalization and the subsequent apoptosis in myotubes and in skeletal muscle from GNE myopathy patients.

## INTRODUCTION

Amyloid β-peptide (Aβ) has received heightened attention in the last years since biomarker studies point to its early involvement in Alzheimer's disease (AD) [1, 2]. In addition, Aβ plays a major role in GNE myopathy, a degenerative muscle disease, characterized by Aβ and other proteinaceous inclusions in skeletal muscle [3, 4].

GNE myopathy is an autosomal recessive disorder characterized by progressive skeletal muscle weakness and wasting but sparing of the quadriceps [5, 6]. It starts in young adulthood and induces disability within 15 years after onset. There is no effective treatment. The disease is also characterized by the presence of atrophic muscle fibers with rimmed vacuoles, filamentous inclusions and intracellular Aβ accumulation [5, 7]. The cause of GNE myopathy is a mutation in the UDP-N-acetylglucosamine 2-epimerase/N-acetylmannosamine kinase (*GNE*) gene [8, 9]. Whereas most of the recessive mutations are missense, patients with biallelic null mutation have not been reported [7, 10]. Moreover, knockout of the *GNE* gene in mice result in embryonic lethality [11], supporting the idea that a certain level of functional protein is required. The *GNE* gene encodes an enzyme that catalyzes the first two rate-limiting steps in the 5-N-acetylneuraminic acid (Neu5Ac) biosynthesis pathway [12-14].

Neu5Ac, usually known as sialic acid, is the most common member of sialic acid family and is precursor to the other members. When mutated, *GNE* encodes a hypofunctional enzyme causing cellular hyposialylation [15-17]. Sialic acids are the most abundant terminal monosaccharides present in glycoproteins and glycolipids, and are mainly located on the cell surface [18]. Interestingly, administration of sialic acid or its precursor ManNAc in GNE myopathy mouse models improves the disease symptoms [19].

Although hyposialylation has been proposed to be the cause of GNE myopathy, the molecular mechanisms that link hyposialylation with intracellular Aβ accumulation and the rest of the pathological features of GNE myopathy remain elusive. Furthermore, the mechanisms responsible for intracellular Aβ toxicity in GNE myopathy are also unknown.

## RESULTS

### Aβ accumulates intracellularly in skeletal muscle cells from GNE myopathy patients

**Figure 1:**
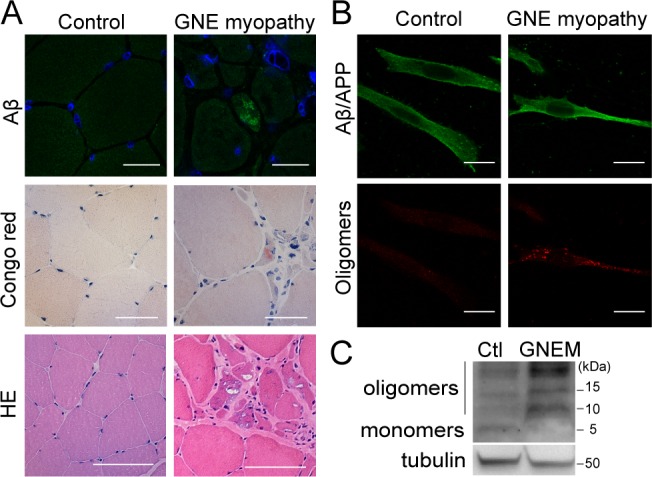
Intracellular Aβ aggregates in GNE myopathy **A.** Representative confocal images of skeletal muscle tissue from a healthy donor and a GNE myopathy patient stained with anti-Aβ (AB5078P antibody) and blue nuclei (Topro-3), scale bar, 25 μm; Congo red, scale bar, 20 μm; and hematoxylin-eosin (HE), scale bar, 50 μm. **B.** Representative confocal images of human myoblasts from a control donor and a GNE myopathy patient stained with anti-Aβ (6E10 antibody); and anti-oligomers (A11 antibody). Scale bar, 50 μm. **C.** Western Blot analysis of Aβ expression in human myoblasts from a control donor and a GNE myopathy patient (GNEM) with 6E10 antibody showing Aβ monomers and oligomers.

We studied Aβ expression within skeletal muscle from a rare GNE myopathy patient. Intracellular Aβ inclusions were detected in several fibers of skeletal muscle sections from the patient, using the anti-Aβ antibody AB5078P (Figure 1A, upper panel), but in none of the 3 age matched controls. Accordingly, positive Congo red staining, a classical method used to detect the presence of fibrillar Aβ, was observed in the same GNE myopathy muscle tissue (Figure 1A, middle panel). The control normal samples were negative. The hematoxylin-eosin stain (Figure 1A, lower panel) evidenced the characteristic atrophic fibers with rimmed vacuoles in GNE myopathy.

Moreover, immortalized myoblasts from a different GNE myopathy patient also showed aggregated Aβ species by immunofluorescence. Using the anti-Aβ 6E10 antibody, a diffuse staining pattern was found in the cytosol and plasma membrane since it also recognizes the Aβ sequence within the amyloid precursor protein (Figure 1B, upper panel). Using the anti-oligomer A11 antibody, discrete accumulations were detected within the cells (Figure 1B, lower panel). Furthermore, enhanced aggregated Aβ forms were observed by Western blot analysis using 6E10 (Figure 1C), where different oligomers were detected and quantified (Figure S1).

### Aβ internalization is enhanced by the hyposialylation of skeletal muscle cells

**Figure 2:**
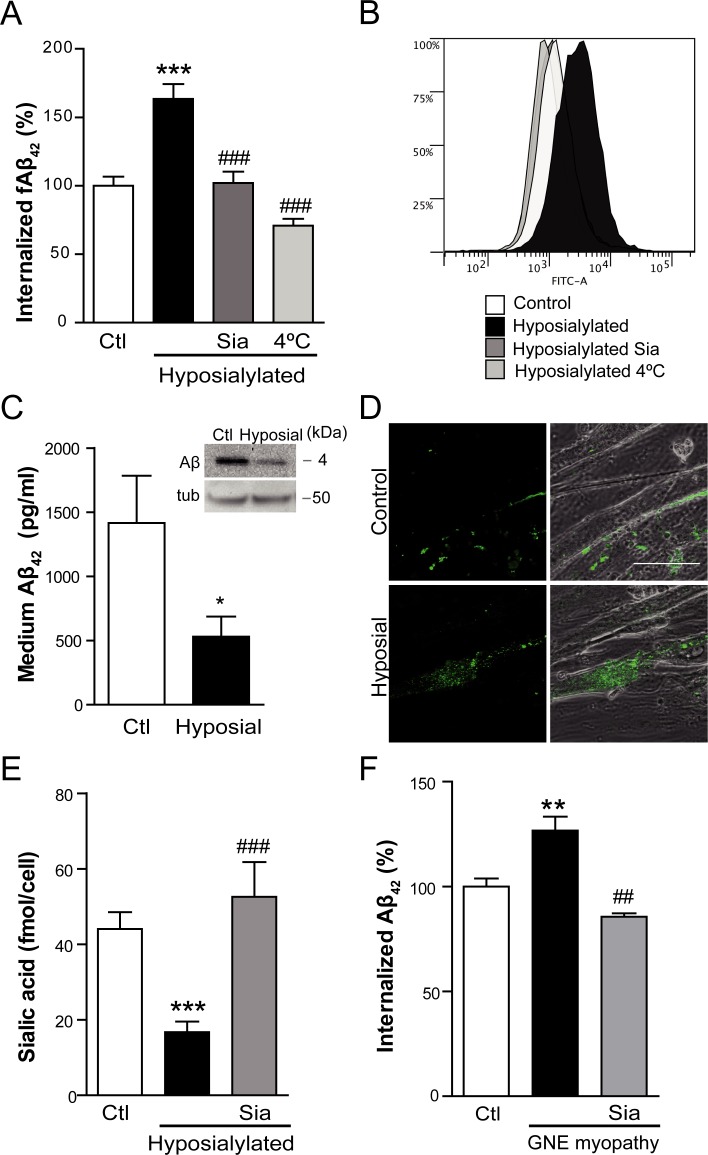
Cell hyposialylation favors Aβ internalization, which is dependent on clathrin and HSPG **A.** Quantification and **B.** representative histogram of intracellular fAβ by flow cytometry in C2C12 myotubes after 2 h of incubation with 250 nM fAβ at the following conditions: control, enzymatically hyposialylated, hyposialylated-resialylated (Sia), and hyposialylated and maintained at 4°C. Data are the mean ± SEM of *n* = 4-17 independent experiments. ***p < 0.001 *vs.* Ctl, ^###^*p* < 0.001 *vs.* Hyposialylated. **C.** Aβ concentration measured by ELISA and Western Blot (inset) in the medium of control and hyposialylated Aβ treated myotubes. Data are the mean ± SEM of *n* = 7 independent experiments performed in triplicate. *p < 0.05. The corresponding cellular tubulin is shown. **D.** Representative confocal images of fAβ uptake in control and hyposialylated myotubes. Scale bar, 50 μm. **E.** Sialic acid concentration measured by the resorcinol periodate method in control, hyposialylated and hyposialylated-resialylated (Sia) myotubes. Data are the mean ± SEM of *n* = 4-9 independent experiments. ***p < 0.001 *vs.* Ctl, ^###^*p* < 0.001 *vs.* Hyposialylated. **F.** Quantification of intracellular fAβ by flow cytometry after 2 h of incubation with 250 nM fAβ of human myoblast from a control, a GNE myopathy patient and the resialylated GNE myopathy myoblasts (Sia). Data are the mean ± SEM of *n* = 3-6 independent experiments. GNE myoblasts are hyposialylated (S2C) **p < 0.01 *vs*. Ctl, ^##^*p* < 0.01 *vs.* GNE myopathy myoblasts.

Since Aβ is produced internally and at the plasma membrane in neurons, undergoing endocytic recycling, before release into the extracellular milieu [20-22], we investigated how Aβ might accumulate in the sarcoplasm in GNE myopathy by focusing on a potential internalization mechanism. To mimic the hyposialylated state found in GNE myopathy, we partially removed the cellular sialic acid levels of C2C12 myotubes with increasing concentrations of *Vibrio Cholerae* neuraminidase (VCN) for 24 h (Figure S2A and Figure 2E). We chose as an experimental model of hyposialylation, the use of 0.03 U/mL of VCN because it produces a significant reduction (*p* < 0.001) of sialic acid concentration in myotubes (Figure S2A).

Aβ internalization was studied by incubating the myotubes with fluorescent tagged Aβ_1-42_ (fAβ) for 2 h and subsequently analyzing intracellular fluorescence by flow cytometry. Aβ uptake was significantly increased in hyposialylated myotubes (*p* < 0.001) (Figure 2A). To confirm that this effect was due to the reduced sialic acid levels and not to any nonspecific action of the VCN treatment, hyposialylated cells were experimentally resialylated by 24 h incubation with Neu5Ac (Sia) (Figure S2B and Figure 2E). As expected, increased Aβ uptake was prevented in resialylated cells (*p* < 0.001) (Figure 2A). Furthermore, when fAβ incubation was done at 4°C, in order to non-specifically inhibit endocytosis processes, the enhanced fAβ internalization detected in hyposialylated myotubes was not observed (Figure 2A). A representative flow cytometry histogram of the data obtained in Figure 2A is shown in Figure 2B.

Similar results were obtained when myotubes were incubated in medium containing non-fluorescent Aβ and the expected change in concentration quantified by ELISA (Figure 2C). Hyposialylation induced a significant decrease (*p* < 0.05) in extracellular Aβ, in agreement with heightened endocytosis. The result correlates with the extracellular Aβ as detected by Western blot (inset in Figure 2C). We also analyzed the internalization of fAβ by confocal microscopy imaging (Figure 2D) finding fAβ aggregates within the hyposialylated but not control, vehicle-treated myotubes. The hyposialylation of the myotubes used in these experiments (and replenishment with Neu5Ac) was confirmed by measuring the cellular sialic acid concentration (Figure 2E), thus mimicking the GNE myopathy condition and our index patient (see below).

Interestingly, identical results such as the ones observed in hyposialylated C2C12 myotubes (Figure 2A and 2B) were obtained when fAβ endocytosis was studied in immortalized myoblasts from a GNE myopathy patient (Figure 2F). These myoblasts have a mutation in exon 7 (*p*.D378Y c.1132G > T) and an allelic mutation in exon 11 (*p*.A631V c.1892C > T) of *GNE* gene (Figure S3) and evidence decreased sialic acid levels (Figure S2C), as reported in previous works [16]. Our data indicate that GNE myopathy myoblasts have a significant higher fAβ uptake (*p* < 0.01; Figure 2F). As before, it can be reverted to normal levels by resialylation (*p* < 0.01). It should be noted that in this case experiments were carried out at the myoblast stage, since these human cells are not able to differentiate into myotubes.

### Aβ is internalized by skeletal muscle cells through clathrin-dependent endocytosis and heparan sulfate proteoglycans (HSPG)

**Figure 3:**
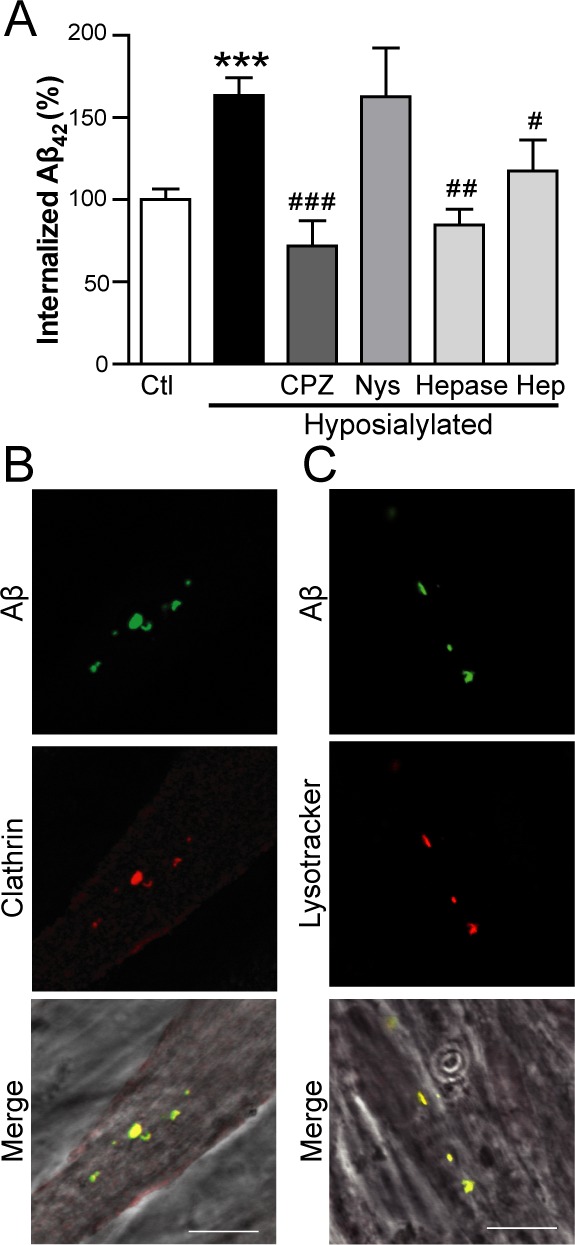
Aβ internalization in hyposialylated C2C12 myotubes is dependent on clathrin and HSPG **A.** Quantification of intracellular fAβ fluorescence by flow cytometry in control and chemically hyposialylated C2C12 myotubes after 2 h incubation with 250 nM fAβ; where indicated, cell cultures were pretreated with 100 μM chlorpromazine hydrochloride (CPZ), 27 μM nystatin (Nys), 5 U/mL heparinase I (Hepase) and 20 μg/mL heparin sodium salt (Hep). Data are the mean ± SEM of *n* = 3-17 independent experiments. ***p < 0.001 *vs.* Ctl, ^#^*p* < 0.05, ^##^*p* < 0.01 ^###^*p* < 0.001, *vs.* Hyposialylated. **B.** Representative confocal images of fAβ colocalisation with clathrin after incubation of C2C12 myotubes with 250 nm fAβ for 2 h. **C.** Representative confocal images of fAβ colocalisation with lysotracker after incubation with 250 nm fAβ for 2 h. Scale bar, 10 μm.

Cellular endocytosis is mainly mediated by clathrin or caveolin pathways. Therefore, in order to further elucidate the mechanism involved in Aβ enhanced internalization, we chemically treated myotubes with specific endocytosis inhibitors for 1 h prior to the 2 h treatment with fAβ (Figure 3A). Our data indicate that fAβ uptake is mediated by the clathrin pathway since the increased fAβ internalization observed in hyposialylated myotubes was prevented by chlorpromazine hydrochloride (*p* < 0.001), an inhibitor of clathrin-mediated endocytosis, but not by nystatin, an inhibitor of caveolin-mediated endocytosis (Figure 3A).

Considering that heparan sulfate proteoglycan (HSPG) has been proposed to play a role in Aβ internalization in different cell types such as human brain vascular smooth muscle cells and human neuroblastoma cells [23, 24], we tested if HSPG was also important in myotubes. As expected, enhanced fAβ endocytosis was also blocked by heparinase I, which selectively cleaves HSPG from cell surface and extracellular matrix, as well as by heparin sodium salt (Figure 3A). These results indicate that the increase in Aβ internalization observed in hyposialylated myotubes is clathrin-mediated and dependent on HSPG.

Accordingly, fAβ was found to colocalize with clathrin within hyposialylated myotubes when cells were incubated with 250 nM fAβ (Figure 3B). The downstream fusion of endocytic vesicles with the lysosomal compartment was studied using lysotracker (Figure 3C). We detected fAβ accumulation in lysotracker-positive acidic vesicles, suggesting that Aβ is directed to the lysosomal pathway after being internalized by myotubes.

### Intracellular Aβ induces apoptosis and cell death in skeletal muscle cells

**Figure 4:**
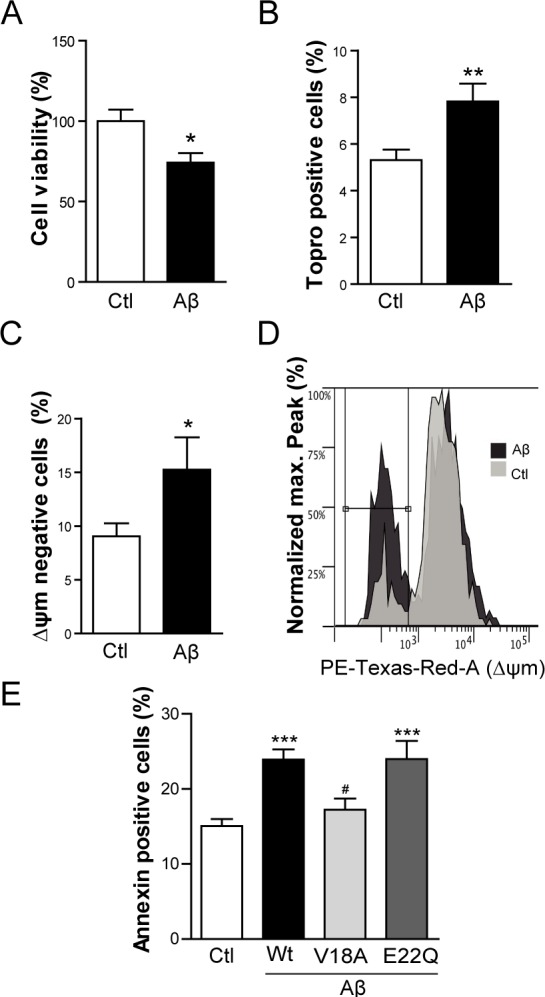
Intracellular Aβ induces cell death and apoptosis in C2C12 myotubes **A.** Cell viability measured by WST-1 reduction in C2C12 myotubes infected with HSV-flit (Ctl) or HSV-Aβ (Aβ). Data are the mean ± SEM of *n* = 10 independent experiments performed in triplicate. *p < 0.05. **B.** Topro-3 positive cell percentage quantification by flow cytometry in myotubes overexpressing GFP (Ctl) or Aβ-GFP (Aβ). Data are the mean ± SEM of *n* = 15-18 independent experiments. **p < 0.01. **C.** Quantification and representative histogram **D.** of the cells with a reduction in mitochondrial membrane potential (Δψm) by flow cytometry in myotubes transfected with GFP (Ctl) or Aβ-GFP (Aβ). Data are the mean ± SEM of *n* = 12-15 independent experiments. *p < 0.05. **E.** Annexin positive cells analyzed by flow cytometry in myotubes overexpressing GFP (Ctl), Aβ-GFP (Wt Aβ), V18AAβ-GFP (V18A Aβ), E22QAβ-GFP (E22Q Aβ). Data are the mean ± SEM of *n* = 7-18 independent experiments. ***p < 0.001 *vs*. Ctl, ^#^*p* < 0.05 *vs.* Wt Aβ.

Extracellular Aβ has been classically associated with neuronal toxicity whereas pathologic effects of intracellular Aβ accumulation are less characterized [25]. To assess the toxicity attributable to the accumulation of intracellular Aβ in skeletal muscle cells, Aβ was transiently overexpressed in C2C12 myotubes using both viral and plasmid methodology. In both cases the signal peptide from amyloid precursor protein was cloned onto the N-terminus of the Aβ sequence in order to direct the expression of the transgene to the endoplasmic reticulum and hence the secretory pathway. Myotubes were either infected with HSV-Aβ_1-42_ or transfected with cDNA encoding Aβ fused to IRES-GFP, which allowed for the identification of Aβ containing cells (GFP positive; Figure S4). By WST assay, myotubes infected with HSV-Aβ showed reduced cell viability compared to cells infected with Ctl HSV (Figure 4A; *p* < 0.05). Non-permeabilized myotubes transfected with Aβ-IRES-GFP were stained using the nuclear marker Topro-3, and the GFP positive population was assessed by flow cytometry. Intracellular Aβ expression again induced toxicity, supported by a modest but significant increase in the percentage of Topro-3 positive cells (*p* < 0.01) among Aβ expressing cells (Figure 4B).

Next, apoptotic events were analyzed by flow cytometry specifically selecting the cell population positive for GFP. Only now, changes in mitochondrial membrane potential were evaluated using MitoTracker Red staining. Aβ overexpressing myotubes showed reduced mitochondrial membrane potential (*p* < 0.05) (Figure 4C and 4D). When cells were stained with Annexin V, which binds to phosphatidylserine that is translocated from the inner to the outer leaflet of the plasma membrane in apoptotic cells, Aβ overexpressing myotubes were Annexin positive relative to control (Figure 4E). The importance of Aβ aggregability was studied using two Aβ mutants cloned into expression plasmid pCAGGSM2-IRES-GFP. The non-aggregative synthetic Aβ mutant (V18A Aβ) [26, 27] failed to trigger apoptosis whereas the highly aggregation prone Aβ mutant (E22Q Aβ) [27, 28] showed approximately the same percentage of Annexin positive cells as the Wild-type Aβ (Wt Aβ) (Figure 4E).

**Figure 5:**
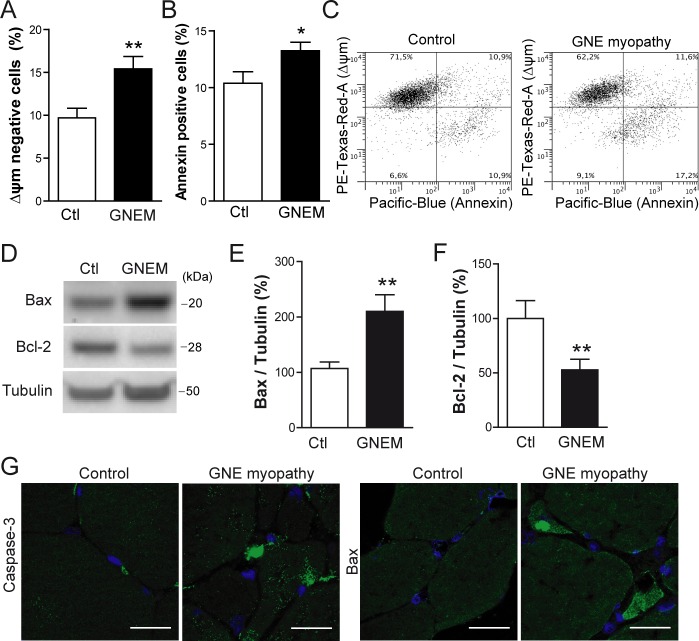
Apoptosis is induced in human myoblasts from GNE myopathy patient **A.** Quantification of cells with reduced mitochondrial membrane potential (Δψm) by flow cytometry in control (Ctl) and GNE myopathy myoblasts (GNEM). Data are the mean ± SEM of *n* = 8-11 independent experiments, **p < 0.01. **B.** Quantification of Annexin positive cell percentage by flow cytometry in control and GNE myopathy (GNEM) myoblasts. Data are the mean ± SEM of *n* = 8-11 independent experiments, *p < 0.05. **C.** Representative plot of data obtained in **A.** and **B.**. **D.** Western blot analysis and densitometric quantification of **E.** Bax and **F.** Bcl-2 in control and GNE myopathy myoblasts. Data are the mean ± SEM of *n* = 10-16 independent experiments, **p < 0.01. **G.** Representative confocal images of cleaved Caspase-3 and Bax expression in skeletal muscle samples from a GNE myopathy patient and a healthy control. Scale bar, 25 μm.

Taking into consideration that GNE myopathy is partly characterized by the accumulation of intracellular Aβ, and the results from viral and plasmid based expression studies above, apoptotic events were assessed in immortalized myoblasts from the GNE myopathy patient and from a control donor. As expected, GNE myopathy myoblasts presented a slight but significant reduction in the mitochondrial membrane potential (Figure 5A and 5C; *p* < 0.01) and an increment in Annexin positive cells (Figure 5B and 5C; *p* < 0.05). Furthermore, expression levels of Bax, a protein that induces cytochrome C release from mitochondria, were markedly increased in GNE myopathy myoblasts compared to control (Figure 5D and 5E; *p* < 0.01). In the same line, the anti-apoptotic Bcl-2 expression levels, were significantly decreased (Figure 5D and 5F; *p* < 0.01). When tissue samples from the GNE myopathy patient were compared to control samples, increased caspase-3 activation and Bax expression levels were detected relative to control, especially within atrophic muscle fibers (Figure 5G).

### Akt activation is impaired by intracellular Aβ aggregates in skeletal muscle cells

**Figure 6:**
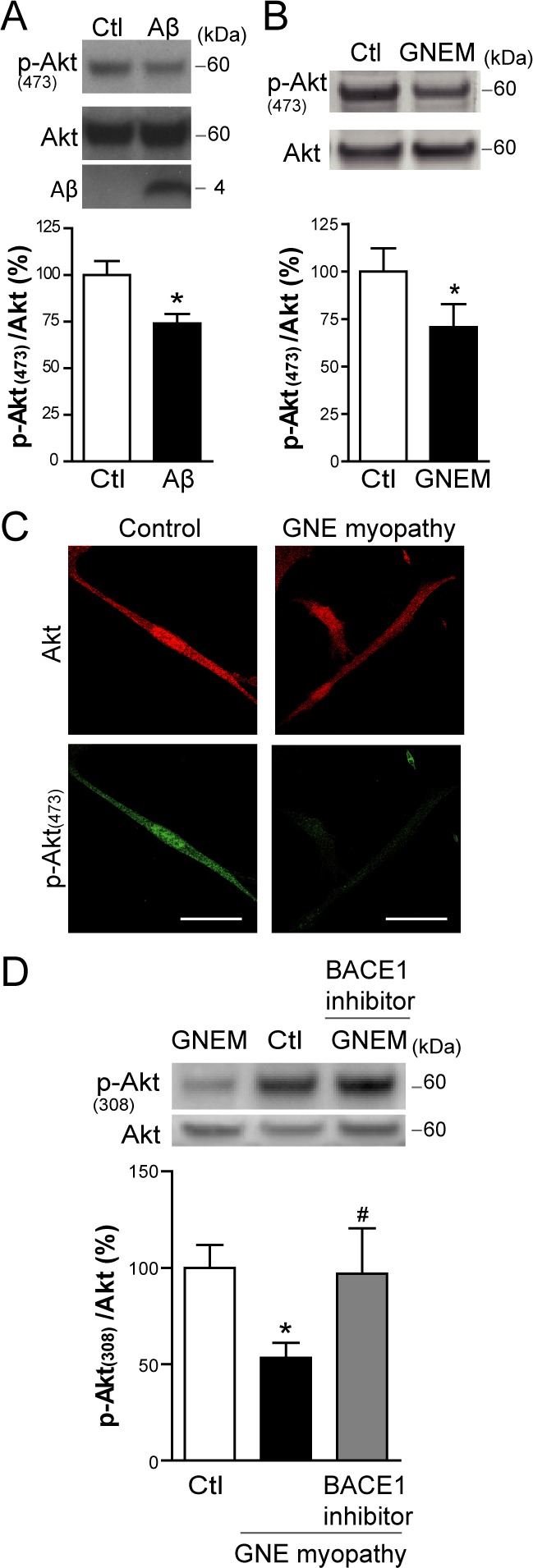
Aβ impairs basal Akt activation in skeletal muscle cells **A.** Western Blot analysis and densitometric quantification of p-Akt (Ser473) and total Akt in C2C12 myotubes infected with HSV-flit (Ctl) or HSV-Aβ (Aβ). Data are the mean ± SEM of *n* = 10 independent experiments, *p < 0.05. **B.** Western Blot analysis and densitometric quantification of p-Akt (Ser473) and total Akt in GNE myopathy (GNEM) and control myoblasts. Data are the mean ± SEM of *n* = 11-14 independent experiments, *p < 0.05. **C.** Representative confocal images of p-Akt (Ser473) in GNE myopathy and control myoblasts. **D.** Western Blot analysis and densitometric quantification of p-Akt (Thr308) and total Akt in control, GNE myopathy myoblasts and GNE myopathy myoblasts pretreated with 1 μM BACE1 inhibitor. Data are the mean ± SEM of *n* = 11-14 independent experiments, *p < 0.05 *vs.* Ctl, # *p* < 0.05 *vs*. GNE myopathy.

During apoptosis, several intracellular survival pathways become deranged. We studied the PI3K/Akt signaling pathway, as reported to be compromised by intracellular Aβ [29, 30]. In the present study, myotubes infected with HSV-Aβ showed a reduction in p-Akt (Ser473) (Figure 6A; *p* < 0.05). Accordingly, we observed by Western Blot (Figure 6B and 6D) and confocal microscopy imaging (Figure 6C) that p-Akt (Thr308 and Ser473) levels were reduced in GNE myopathy myoblasts compared to control myoblasts (*p* < 0.05). The Akt impairment detected in GNE myopathy myoblasts is likely mediated by Aβ since it was prevented by a β-secretase BACE1 inhibitor, which inhibits cellular Aβ generation (Figure 6D).

**Figure 7:**
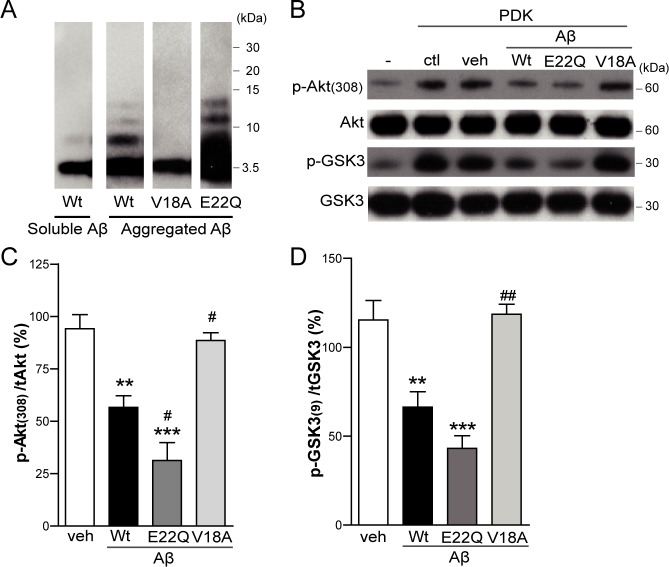
Aβ impairs Akt activation in *in vitro* studies Inactive human recombinant Akt was incubated with GSK3 fusion protein and, where indicated, with active human recombinant PDK-1 protein plus DMSO (veh), Wt Aβ, V18A Aβ or E22Q Aβ synthetic peptides. **A.** Western Blot analysis of *in vitro* preparation of synthetic wild type (Wt) Aβ, V18A Aβ and E22Q Aβ oligomeric aggregates compared to soluble Wt Aβ monomers. Western Blot analysis **B.** and densitometric quantification (**C**. and **D**.) of p-Akt (Thr308) and p-GSK3 (Ser21/9). Data are the mean ± SEM of *n* = 3-4 independent experiments, **p < 0.01, ***p < 0.001 *vs.* veh, ^#^*p* < 0.05, ^##^*p* < 0.01 *vs.* Wt Aβ.

To further address Aβ effect, and its dependence on the aggregate state (Figure 7A), on enzymatic activity itself, a cell free *in vitro* Akt assay was performed. Recombinant human active phosphoinositide-dependent kinase-1 (PDK-1) was incubated with recombinant human inactive Akt and with GSK3 fusion protein, as the Akt substrate. Basal phosphorylation levels of Akt and GSK3 were observed, which markedly increased when PDK-1 was added to the reaction mixture, as expected (Figure 7B). When synthetic Aβ was coincubated in the mixture, Akt and GSK3 phosphorylations were inhibited (*p* < 0.01), indicating an impairment in Akt activation, as previously reported in a similar cell-free assay [31]. In addition, the relevance of Aβ aggregation in the impairment of Akt activation was studied. For that purpose, the highly aggregative E22Q Aβ mutant and the non-aggregative V18A Aβ mutant were added to the mixture. E22Q Aβ inhibited Akt phosphorylation to a higher degree than Wt Aβ (*p* < 0.05) whereas V18A Aβ had no effect on Akt activation, similar to the vehicle control (Figure 7B and 7C). Confirmatory results pertinent to enzymatic activity were obtained when GSK3 phosphorylation was analyzed (Figure 7B and 7D). These data suggest that Aβ inhibits Akt activation in an aggregation dependent manner.

**Figure 8:**
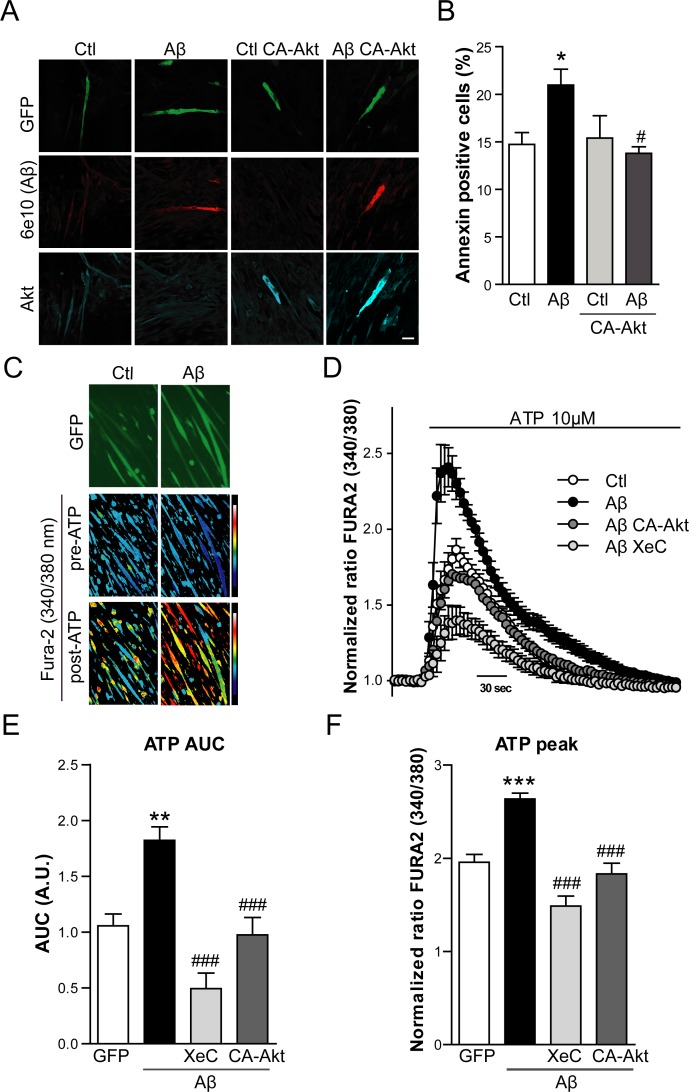
Aβ induced apoptosis and enhanced IP_3_R-mediated Ca^2+^ release is prevented by Akt overexpression **A.** Representative confocal images of C2C12 myotubes overexpressing GFP (Ctl), Aβ-GFP (Aβ), GFP and CA-Akt (Ctl CA-Akt) and Aβ-GFP and CA-Akt (Aβ CA-Akt). Cells were stained with anti-Aβ (6E10 antibody) and with anti-Akt antibody. Scale bar, 50 μm. **B.** Quantification of Annexin positive cell percentage by flow cytometry in myotubes transfected as described in **A.** Data are the mean ± SEM of *n* = 8-9 independent experiments, *p < 0.05 *vs.* Ctl, ^#^*p* < 0.05 *vs.* Aβ. **C.** Ratiometric images of Fura-2/AM-loaded myotubes overexpressing GFP (Ctl) and Aβ-GFP (Aβ), before and after 10 μM ATP application. Color scale shows the pseudocolor coding of ratio values ranging from low (blue) to high (white). **D.** Time course of mean Ca^2+^ responses (Fura-2 normalized ratio) obtained in myotubes overexpressing GFP (Ctl), Aβ-GFP (Aβ), Aβ-GFP and CA-Akt (Aβ CA-Akt) and Aβ-GFP pretreated with Xestospongin C (Aβ XeC), after a 10 μM ATP stimuli in the absence of Ca^2+^ in the extracellular solution. Data are the mean ± SEM of *n* = 7-16 independent experiments. Quantification of the AUC **E.** and Average peak [Ca^2+^] increases **F.** obtained from traces shown in **D.**. Data are the mean ± SEM of *n* = 7-16 independent experiments, **p < 0.01, ***p < 0.001 *vs.* Ctl, ^###^*p* < 0.001 *vs.* Aβ.

Subsequently, the role of Akt activation impairment was evaluated as a potential mechanism of Aβ toxicity in the present experimental model. Myotubes were cotransfected with Aβ-IRES-GFP and a constitutively active form of Akt (CA-Akt) and the expression of the transgenes was confirmed by confocal microscopy imaging (Figure 8A). Interestingly, when CA-Akt was overexpressed, the apoptosis induced by Aβ was prevented (*p* < 0.05) (Figure 8B), since Annexin positive cell percentage returned to the control level.

### Intracellular Aβ enhances IP_3_R-mediated Ca^2+^ release

Akt is known to inhibit inositol 1,4,5-trisphosphate receptor (IP_3_R) mediated Ca^2+^ release and to prevent the consequent induction of apoptosis [32-34]. On the other hand, Aβ cytotoxicity involves Ca^2+^ dysregulation in AD and in particular, Aβ is claimed to impair endoplasmic reticulum Ca^2+^ homeostasis in several cell types [35]. Taking into consideration these facts, we measured IP_3_R mediated Ca^2+^ release in C2C12 myotubes overexpressing Aβ.

The fluorescent indicator Fura-2/AM was used to measure cytosolic Ca^2+^ concentration in Aβ containing myotubes identified by GFP fluorescence (Figure 8C). In order to quantify Ca^2+^ release into the cytosol through the IP_3_R in the sarcoplasmic reticulum, ATP was added to the myotubes. ATP activates P2U-purinergic receptors triggering the formation of IP_3_, which activates IP_3_R and Ca^2+^ release from the sarcoplasmic reticulum [36]. To rule out a confounding effect of extracellular Ca^2+^, ATP stimuli was done under calcium-free conditions. Treatment with ATP induced a rapid cytosolic Ca^2+^ transient which was enhanced in Aβ overexpressing myotubes (Figure 8D). This response was mostly prevented by pretreating cells with Xestospongin C, an inhibitor of IP_3_R. Interestingly, in myotubes cotransfected with the CA-Akt, the increased Ca^2+^ transient induced by ATP in Aβ overexpressing cells was significantly prevented, suggesting that Akt is involved in the effect that Aβ exerts in the IP_3_R-mediated Ca^2+^ release. Area under the curve and average peak [Ca^2+^] increases obtained from traces shown in Figure 8D were quantified in Figure 8E and 8F, respectively.

## DISCUSSION

It is well known that the cause of GNE myopathy is a mutation in the *GNE* gene that produces a decrease in the sialic acid content in skeletal muscle cells [15, 16]. Furthermore, GNE myopathy is noted for the accumulation of intracellular Aβ [4, 37]. However, to our knowledge, the precise molecular mechanisms that link hyposialylation with Aβ accumulation remain elusive.

To address the first objective, Aβ internalization was examined in C2C12 myotubes and this study reports for the first time that Aβ can be endocytosed by skeletal muscle cells. In order to mimic the condition of reduced sialic acid content found in GNE myopathy, myotubes were treated with VCN, which decreases the cellular level of sialic acid. The results demonstrate that in hyposialylated myotubes incubated with Aβ, intracellular levels accumulate whereas the amount of Aβ in the medium was correspondingly decreased compared to control myotubes; supporting the notion that hyposialylation favors Aβ internalization and deposition in GNE myopathy. Furthermore, when sialic acid is added back to hyposialylated cells to levels approximating the normal condition, the enhanced internalization does not occur. In the same line, Aβ internalization was increased in immortalized human myoblasts from the GNE myopathy patient, effect that was prevented by resialylation. This fits with the clinical recovery of the GNE myopathy mouse model after sialic acid administration [19].

A possible explanation for this effect is that sialic acid is moderating Aβ endocytosis in control conditions, probably because Aβ gets directly attached to sialic acid [38] leading to retention and aggregation in the extracellular matrix [39]. Alternatively, when there is a reduction in the sialic acid content of the cell membrane, Aβ may have access to other receptors or transporting molecules. Some of these could also be modified by sialic acid, leading to Aβ internalization.

We further investigated mechanisms of Aβ endocytosis. The fact that the clathrin-mediated endocytosis inhibitor, chlorpromazine hydrochloride but not the caveolin-mediated endocytosis inhibitor, nystatin, prevented the enhanced Aβ internalization, indicates that it is dependent on clathrin-mediated endocytosis. Accordingly, there is a report showing increased clathrin granules in muscle from patients with GNE myopathy [40]. Another key molecule that specifically regulates Aβ endocytosis in neuroblastoma cells, human brain vascular smooth cells and astrocytes is HSPG [24, 41]. Here it is shown that HSPG is also involved in the enhanced Aβ internalization observed in hyposialylated myotubes since when cells are treated with heparin or heparinase-I, the enhanced internalization is prevented.

In order to address the consequences of enhanced Aβ endocytosis in skeletal muscle cells, Aβ was overexpressed within myotubes. Our results point that intracellular Aβ triggers cell death and apoptotic events in myotubes, including enhanced Annexin positive cell percentage and reduced mitochondrial membrane potential, in agreement with previous reports [42]. The aggregation status of Aβ seems to be crucial for the induction of apoptosis. When a non-aggregative mutant of Aβ (V18A Aβ) [26, 27] is overexpressed, apoptosis is not induced suggesting that intracellular Aβ monomers are not toxic to cells.

Accordingly to our data, apoptotic events appear to be induced in human immortalized myoblasts from a GNE myopathy patient. Evidence for this shown here for the first time includes: enhanced Annexin positive cell percentage, reduced mitochondrial potential membrane, increased pro-apoptotic Bax expression levels and decreased anti-apoptotic Bcl-2 protein levels in GNE myopathy myoblasts. A quite recent study evaluating apoptotic events in primary myoblast culture from GNE myopathy patients demonstrates enhanced active Caspase-3 and Caspase-9 in myoblast from patients [43]. Our results on skeletal muscle sections from the GNE myopathy patient confirm this by demonstrating an increase in active Caspase-3 and Bax expression.

The present results, also in agreement with previous reports [30, 31], demonstrate that intracellular Aβ impairs Akt activation, a major anti-apoptotic kinase [44, 45]. Both systems used for Aβ overexpression, transduction and transfection, show that intracellular Aβ accumulation inhibits Akt activation since its phosphorylation level is decreased. In particular, a cell free *in vitro* Akt assay evidences that Aβ clearly inhibits PDK-1 mediated Akt activation. This effect was already reported using immunoprecipitated Akt and PDK-1 [46], whereas the assay here was performed using recombinant inactive Akt and recombinant active PDK-1 in an attempt to avoid any cellular interference. Furthermore, we provide evidence that Akt inhibition by Aβ is dependent on the aggregation properties of the peptide since the non-aggregative mutant V18A Aβ has no effect on Akt phosphorylation. This is in accordance with the toxicity data showing that V18A Aβ does not induce apoptosis in myotubes. These observations support the hypothesis that Akt inhibition constitutes a critical mechanism of Aβ cellular toxicity. In fact, we show that Aβ induced apoptosis can be prevented by overexpressing a CA-Akt, as reported in primary neuronal cultures [30].

Finally, the specific molecular mechanism that triggers apoptosis through Aβ induced Akt inhibition was further assessed. Akt is known to inhibit IP_3_R mediated Ca^2+^ release from the ER preventing apoptotic events [32, 33]. In contrast, Aβ cytotoxicity has been related to Ca^2+^ dysregulation in AD and more specifically, to dysfunctional ER Ca^2+^ signaling [35, 47]. For instance, fibroblasts from asymptomatic patients at risk for AD show an enhanced IP_3_R-mediated Ca^2+^ signaling [48]. We proved that Aβ accumulation causes an increase in ATP-dependent cytosolic Ca^2+^ release, mediated by IP_3_R. This is one of several mechanisms that could lead to apoptosis [32, 49]. Enhanced Ca^2+^ transients following addition of ATP to Aβ containing myotubes, were prevented by Xestospongin C, an inhibitor of IP_3_R. The response did not require extracellular Ca^2+^, further pointing toward mediation by this receptor. Furthermore, the increase in cytosolic Ca^2+^ observed in Aβ overexpressing myotubes did not occur when cells co-expressed constitutively active Akt. This indicates that the Aβ-induced, IP_3_R release effect is mediated, at least partially, by Akt inhibition. It is consistent with the regulation of IP_3_R by PKB1/Akt [32]. Our proposed model consists of hyposialylation leading to Aβ accumulation, Aβ inhibiting Akt activation with consequent loss of IP_3_R regulation by Akt, resulting in an increase of Ca^2+^ released from the ER and finally to apoptosis (Figure S5).

To conclude, the present report provides for the first time compelling evidence that a reduction in sialic acid, condition found in GNE myopathy, favors Aβ internalization in skeletal muscle cells. This supports the pharmacological therapeutic approaches based on sialic acid administration for GNE myopathy [50, 51]. Furthermore, our findings describe enhanced apoptotic events in skeletal muscle from GNE myopathy patients as well as in myotubes overexpressing Aβ. This apoptosis seems to be caused by an impairment of Akt phosphorylation produced by Aβ and consequent dysfunction in Ca^2+^ homeostasis. Our observation reinforces the premise that mitigating Aβ effect on Akt activation could constitute a therapeutic approach for GNE myopathy as well as AD or sporadic inclusion body myositis, which are all characterized by intracellular Aβ accumulation.

## MATERIALS AND METHODS

### Human samples

A muscle biopsy was performed for diagnostic purposes on a 34-year-old woman suffering from GNE myopathy. Full consent was obtained for subsequent use of the sample for this project under University Hospital protocol. Biopsies from three age-matched normal controls (non family members) were collected for the present study. The samples were obtained after informed consent and kept at −80°C.

### Cell cultures

C2C12 myoblasts were maintained in growth medium consisting of Dulbecco's modified essential medium supplemented with 20% Fetal Bovine Serum (Life Technologies). To induce differentiation, myoblasts were grown to confluence and then growth medium was replaced by differentiation medium containing 2% heat inactivated Horse Serum (Life Technologies). Differentiation medium was changed every two days during 3-6 days when most of the myoblasts had fused. Human myoblasts immortalized from a healthy donor and from a GNE myopathy patient with a mutation in exon 7 (*p*.D378Y c.1132G > T) and a mutation in exon 11 (*p*.A631V c.1892C > T) of *GNE* gene (kindly gifted by Darvish D and Valles-Ayoub Y., Reseda, USA), were grown in Ham's F10 medium supplemented with 0.5% chick embryo extract (Sera Laboratories) and 15% FBS. When indicated, cells were incubated for 24 h with 1 μM BACE1 inhibitor compound IV (Merck). All chemicals were obtained from Sigma-Aldrich unless otherwise noted.

### Sialic acid quantification

Cells were scrapped with PBS and counted. After three washes with PBS, cells were resuspended with 250 μL PBS and lysed by 4 cycles of freezing and thawing. The samples were oxidized with 5 μL periodic acid 0.4 M and incubated 1.5 h at 4°C, then 500 μL of 6% resorcinol, 2.5 mM CuSO_4_, 44% HCl were added and the samples were boiled for 15 min. After that, the samples were cooled under running tap water and 500 μL t-butyl alcohol were added. After vortexing, samples were centrifuged 15700 g for 5 min, the supernatants were transferred to a cuvette and optic density was measured at 630 nm. Sialic acid concentrations were calculated by comparison of these optical density values with a standard curve generated from known concentrations of sialic acid.

### Aβ_1-42_ internalization

Myotubes and myoblast internalization of HiLyte Fluor488 labeled human Aβ_1-42_ (fAβ_1-42_) (Anaspec) was quantified by flow cytometry. Cell cultures were exposed to 250 nM fAβ_1-42_ in serum free Ultraculture medium for 2 h or 24 h. When specified, 0.03U/mL VCN (Invitrogen) was added 24 h before fAβ_1-42_. Cell cultures were treated with the following inhibitors 2 h before adding fAβ_1-42_: 100 μM chlorpromazine hydrochloride, 20 μg/mL heparin sodium salt, 27 μM nystatin and 5 U/mL heparinase I from *Flavobacterium heparinum*. After 4 washes with PBS, cell cultures were incubated with trypsin for 15 min in order to eliminate extracellular binding of fAβ_1-42_. Cells were collected with Ultraculture medium and centrifuged for 5 min at 300 g at 4°C, washed with cold PBS and resuspended and incubated with Propidium Iodide 5 min at room temperature (RT). fAβ_1-42_ positive and Propidium Iodide negative cells were quantified using the FACS FORTESSA cytometer and analyzed by the FlowLogic Software. Myotubes were gated based on morphological appearance (forward and side scatter) and the same gates were used for all experiments. Internalization of fAβ_1-42_ was expressed as the mean fluorescence intensity of Fluor488 in the FITC-A channel. In each experiment non fAβ_1-42_ treated cells were used as reference for the mean fluorescence intensity quantification.

Myotubes were also exposed to 250 nM of non-fluorescent Aβ_1-42_ (Anaspec) for 24 h, conditioned medium was collected and cells were lysed. The levels of Aβ_1-42_ in the medium were measured using commercial enzyme-linked assay kits (IBL, Gumna) as previously described [52] and Western Blot technique using mouse anti-Aβ 6E10 1:600 (Covance).

### Cell viability assay

Cell viability was measured by 4-[3-(4-Iodophenyl)-2-(4-nitrophenyl)-2H-5-tetrazolio]-1,3-benzene disulfonate (WST-1, Roche) reduction. WST-1 solution was added to the cells and after 4 h, reduced WST-1 was determined at 490 nm in a Vmax microplate reader (Molecular Device, Sunnyvale, CA, USA).

### Apoptosis analysis

Control and transfected or treated cells were incubated with staining solution containing 400 nM MitoTracker Red CMXRos (Life Sciences) for 45 minutes. Then, cells were trypsinized, washed in PBS and resuspended in Annexin-binding buffer. Annexin V Pacific Blue conjugate and Topro-3 (Life Sciences) were added to each cell suspension and incubated for 15 min at RT. Excess of Annexin-binding buffer was added and cells were quantified using the FACS FORTESSA cytometer and analyzed by the FlowLogic Software. When transfected cells were analyzed, only GFP positive population was considered.

### Synthetic Aβ peptides

Monomeric Aβ_1-42_, V18A Aβ_1-42_ and E22Q Aβ_1-42_ (EzBiolabs and Anaspec) were prepared dissolving the peptide in 1,1,1,3,3,3-hexafluoro-2-propanol, evaporated and resuspended in DMSO or dissolving the peptide in 1M NaOH pH 10.5, diluted to 0.443 mM with 20 mM Phosphate Buffer pH 7.5 and sonicated 3 times for 1 min. In order to obtain the oligomeric Aβ-derived diffusible ligands, the monomeric-enriched Aβ fraction was diluted to 100 μM in F12 medium lacking phenol red and incubated at 4°C for 24 h. All peptide preparations were used fresh or flash-frozen and stored at −80°C until use.

### *In vitro* Akt assay

10 ng of active human recombinant PDK-1 protein (Amsbio) and 100 ng of inactive human recombinant Akt1 (Amsbio) were mixed in a reaction containing kinase buffer (25 mM Tris, pH 7.5, 2 mM DTT, 0.1 mM Na_3_VO_4_, 10 mM MgCl_2_, and 200 μM ATP), 50 nM PIP_3_, 300 μM ATP, 10 μM Aβ_1-42_, V18A Aβ_1-42_, E22Q Aβ_1-42_ or the vehicle and H_2_O until 50 μl of total volume. After mixing well, the reaction was incubated 15 min at 30°C and then 0.5 μM of GSK-3 Fusion protein (Cell Signaling) was added and incubated for 20 min at 30°C. The reaction was ended by adding Laemmli loading buffer and after boiling the samples 10 min at 95°C, samples were resolved in 4-12% Bis-Tris Gel (Invitrogen).

### mRNA extraction and RT-PCR

RNA extraction (Nucleospin RNA II kit, Macherey-Nagel) was carried out and RT-PCR was performed using SuperScrip-RT (Invitrogen). Aliquots of 1 μg cDNA aliquots were used as template for PCR. The primers used to sequencing GNE from human myoblasts were 5′- TATGGGGATGGAAATGCTGT-3′, 5′-TATTGCAACTCGGAGGTTCG-3′ for exon 7 and 5′-AAGCATACGCCTCTGGAATG-3′, 5′-GATCACAAGGGAGGGATTCA-3′ for exon 11.

### Cloning and mutagenesis of human Aβ

The Aβ_1-42_ sequence of the amyloid precursor protein gene was amplified from the cDNA of the SH-SY5Y cell line using the following primers: 5′-CGGATCCATGGATGCAGAATTCCGACATG-3′, 5′-CACGCGTCTACGCTATGACAACACCGC-3′. The PCR product was purified from an agarose gel using the Ilustra™ GFX™ PCR DNA and Gel Band Purification kit (GE Healthcare) and inserted into the pTZ57R/T vector (Fermentas). cDNA was subcloned into the BamHI and XbaI sites of the vector pcDNA3.1. For the site-directed mutagenesis we used the QuikChange Mutagenesis Kit (Stratagene). For the V18A Aβ_1-42_ the valine in position 18 was mutated to alanine using the following primers 5′-CATCATCAAAAATTGGCGTTCTTTGCAGAAG-3′, 5′-CTTCTGCAAAGAACGCCAATTTTTGATGATG-3′ and for the E22Q Aβ_1-42_ (Dutch) the glutamic acid in position 22 was mutated to glutamine using the primers 5′-CAAAAATTGGTGTTCTTTGCACAAGATGTGG-3′, 5′-CCACATCTTGTGCAAAGAACACCAATTTTTG-3′. The Aβ_1-42_ construct was then modified with an additional N-terminal signal peptide (SP) of the amyloid precursor protein and a bridging DA dipeptide. The SP sequence was amplified from SH-SY5Y cDNA with the following primers 5′-CGGATCCATGCTGCCCGGTTTGGCACTG-3′,5′-TCGGAATTCTGCATCAGCATCCGCCCGAGCCGTCCAGG-3′ and the Aβ_1-42_ sequence was amplified from the PTZ vector using the primers5′-GGCTCGGGCGGATGCTGATGCAGAATTCCGACATGACTCAGGA-3′, 5′-CACGCGTCTACGCTATGACAACACCGCCCA-3′. An overlapping PCR was used to obtain the SPAβ_1-42_ sequence (CGGATCCATGCTGCCCGGTTT GGCACTGCTCCTGCTGGCCGCCTGGA CGGCTCGGGCAGTTCATCATC AAAAATTGGTGTTCTTTGCAGAA GATGTGGGTTCAAACAAA GGTGCAATCATTGGACTCATGGT GGGCGGTGTTGTCATA GCGTAGACGCGTG) which was then inserted into the PTZ vector and subcloned into the BamHI sites of vector pcDNA3.1 as well as downstream the actin promotor into the SacI and XhoI site of pCAGGSM2-IRESGFP vector (kindly gifted by Nilius B., KU Leuven, Belgium). All constructs were verified by sequentiation (Big Dye 3.1, AbiPrism, Applied Biosystems). The construct obtained SPAβ-IRESGFP was used in all Aβ_1-42_ overexpression experiments. In Akt overexpression experiments the control plasmid pIRESGFP or the plasmid with the constitutively active form of Akt CA-Aktp-IRESGFP was used (a kindly gift from Malagelada C., Barcelona, Spain).

### Aβ_1-42_ overexpression

C2C12 myotubes were transiently transfected with the Aβ construct (SPAβ-IRESGFP, as described above). A total of 1 μg of DNA was transfected into each well of a 24 well plate using 1 μL of Lipofectamine 3000 (Invitrogen) according to the manufacturer's instructions. After 12 h, medium was changed to differentiation medium and cells were incubated for 3 additional days. In some experiments myotubes were infected with herpes simplex virus vector encoding Aβ_1-42_ (HSV-Aβ) and FLAG-tagged Flt (HSV-ctl) at a multiplicity of infection of 1.0 and cultivated for 36 h. Flt is a 44 amino acid peptide corresponding to the VEGF-R transmembrane domain serving as a control peptide. Synthesis and preparation of HSV stocks were performed in the MIT Viral Gene Transfer Core (Dr. Rachael Neve, Ph.D., Director).

### Protein levels detection by western blot

Cells were lysed with 1% Triton buffer (150 mM NaCl, 1 mM EDTA, 1mM EGTA 1% Triton X-100, 1 mM sodium orthovanadate, 2.5 mM sodium pyrophosphate, 1 mM β-glycerophosphate, 1 μg/mL leupeptin, 0.1 mM phenylmethylsulphonyl fluoride, 20 Mm Tris pH 7.5) and 1x of protease inhibitors (Complete mini-EDTA free, Roche Diagnostics). The lysate was centrifuged at 10000 x g for 10 min and the protein concentration was measured by BCA assay. Protein (8-100 μg) was mixed with LDS Sample Buffer (4X) and Sample Reducing Agent (10X) (Life Technologies) and incubated 10 min at 70°C. Samples were resolved in 4-12% Bis-Tris Gel (Life Technologies). Gels were transferred in polyvinylidene fluoride membranes (ImmobilonP, Millipore). The membranes used for Aβ detection were incubated for 5 minutes in boiled PBS. Membranes were exposed for 1 h to a blocking solution containing tween-tris buffer saline 5% milk or 3% Albumine from Bovine Serum. Membranes were incubated overnight at 4°C with the following primary antibodies: mouse anti-tubulin 1:4000 (Sigma), rabbit anti-Bax 1:700, rabbit anti-Bcl2 1:500, mouse and rabbit anti-phospho-Akt (Thr308 and Ser473) 1:1000, mouse and rabbit anti-Akt 1:1000, rabbit anti-p-GSK3β (Ser21/9), mouse anti-GSK3β (Cell Signaling) and mouse anti-Aβ 6E10 1:300 (Covance). After three washes with tween-tris buffer saline, membranes were incubated for 1 h with anti-mouse or anti-rabbit secondary antibodies (GE-Healthcare) at 1:2000 dilutions. Five washes with tween-tris buffer saline were performed and membranes were developed with Supersignal West Pico and Femto Chemiluminiscent substrate (Thermo Scientific Pierce). Blotting quantification was done with Quantity One software.

### Cell immunofluorescence experiments

C2C12 myoblasts were seeded on gelatin coated coverslips and differentiated as previously described. Lysotracker 75 nM was added for 45 min when specified. After two washes with PBS, cells were fixed with 4% paraformaldehyde for 10 min. After three PBS washes, cells were permeabilized with 0.1% Triton X-100 and washed thrice with PBS. Coverslips were incubated with blocking solution (5% Fetal Bovine Serum, 1% Albumine from Bovine Serum and 0.02% sodium azide) overnight at 4°C. Subsequently, cells were incubated for 2 h at RT in a hydration chamber with the following primary antibodies: mouse anti-Aβ 6E10 1:200, rabbit anti-oligomers A11 1:200 (Invitrogen), mouse anti-phospho-Akt Ser473 1:100, rabbit anti-Akt 1:100 and mouse anti-clathrin heavy chain 1:200 (BD Biosciences). Then, cells were washed thrice with blocking buffer and incubated with Alexa Fluor 488 or 555 goat anti-mouse antibodies (Invitrogen) 1:1000 for 1 h at RT. When specified, cells were incubated with 1μM Topro-3 for 15 min, washed with PBS and mounted with Mowiol. Digital images were taken at RT with a Leica TCS SP confocal microscope with ×40 and ×63 objective and analyzed with Leica confocal software (Heidelberg, Germany) and Image J software.

### Histological stains

Muscle cryostat sections 8 μm-thick were stained with hematoxylin and eosin (H&E) and amyloid-like deposits were visualized using Congo red stains.

### Immunohistofluorescence experiments

Muscle cryostat sections 8 μm-thick were incubated with blocking solution (0,25% Triton X-100, 3% Albumine from Bovine Serum in PBS) for 1h at RT. Subsequently, cells were incubated for 2 h at RT in a hydration chamber with the following primary antibodies at 1:50 dilution: rabbit anti-Aβ AB5078P (Millipore), rabbit anti-Bax and rabbit anti-cleaved Caspase3 (Abcam). Then, cells were washed thrice with PBS and incubated with Alexa Fluor 488 or 555 goat anti-mouse antibodies 1:500 for 2 h at RT. After 3 PBS washes, cells were incubated with 1μM Topro-3 for 10 min, washed with PBS and mounted with Mowiol. Digital images were taken at RT with a Leica TCS SP confocal microscope with ×40 objective and analyzed with Leica confocal software.

### Measurement of intracellular [Ca^2+^] in C2C12 myotubes

Cytosolic Ca^2+^ signal was determined at RT in cells loaded for 45 min with 4.5 μM FURA 2-AM (Life Technologies). Calcium measurements were performed on a NIKON inverted microscope at RT with a ×20 objective. Fura-2 ratiometric images were acquired every 2 s with a digital camera (Hamamatsu Photonics) and analyzed with the AquaCosmos software. Cytosolic [Ca^2+^] increases are presented as the ratio of emitted fluorescence (510 nm) after excitation at 340 and 380 nm (340/380 ratio), relative to the ratio measured prior to cell stimulation (F/Fo). During all experiments cells were bathed in an isotonic normal Tyrode's solution containing (in mM): 137 NaCl, 5 KCl, 1.8 CaCl_2_, 0.5 MgCl_2_, 5.55 glucose, 11.8 HEPES (300 mosmol/L, pH 7.4 with Tris). 5 min before and during ATP application, cells were incubated with 0 Ca^2+^ Tyrode's solution containing (in mM): 137 NaCl, 5 KCl, 2.3 MgCl_2_, 5.55 glucose, 11.8 HEPES, 2.5 EGTA (300 mosmol/L, pH 7.4 with Tris).

### Statistical analysis

Data are expressed as mean ± SEM of n independent experiments. Statistical analyses were performed by using One-way ANOVA followed by Bonferroni's post hoc analysis for more than two sets of data or the Student unpaired *t* test for two sets of data.

## SUPPLEMENTARY MATERIAL FIGURES



## Abbreviations

Aβ: amyloid β-peptide
AD: Alzheimer's disease
CA-Akt: constitutively active form Akt
fAβ: fluorescent tagged Aβ_1-42_
GNE: UDP-N-acetylglucosamine 2-epimerase/N-acetylmannosamine kinase
HSPG: heparan sulfate proteoglycans
IP3R: inositol 1,4,5-trisphosphate receptor
Neu5Ac: 5-N-acetylneuraminic acid
PDK-1: phosphoinositide-dependent kinase-1
VCN: *Vibrio Cholerae* neuraminidase.
