# Supplementary material for: Increased amyloid β-peptide uptake in skeletal muscle is induced by hyposialylation and may account for apoptosis in GNE myopathy
Source: Oncotarget. 2016 Mar 8;7(12):13354–71. doi: 10.18632/oncotarget.7997 (PMC4924647; doi:10.18632/oncotarget.7997)
Supplement: Supplementary file 1 [file oncotarget-07-13354-s001.pdf]

Increased amyloid β-peptide uptake in skeletal muscle is induced by hyposialylation and may account for apoptosis in GNE myopathy

Supplementary Material

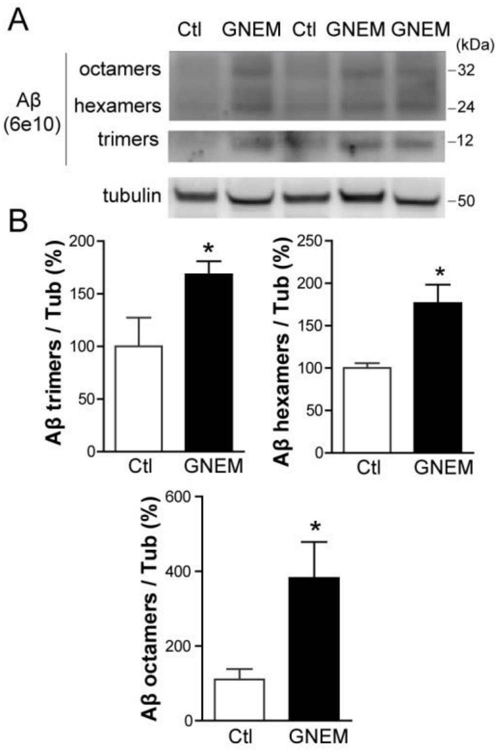

Supplementary Figure S1. Intracellular Aβ expression in GNE myopathy. **(A)** Western Blot analysis and **(B)** densitometric quantification of oligomeric Aβ expression in immortalized myoblasts from a control donor and from a patient with GNE myopathy using 6E10 antibody. 12 kDa band corresponds to Aβ trimers, 24 kDa to Aβ hexamers and 32 kDa to Aβ octamers. Data are the mean ± SEM of n = 3-5 independent experiments. *p<0.05 *vs.* control.

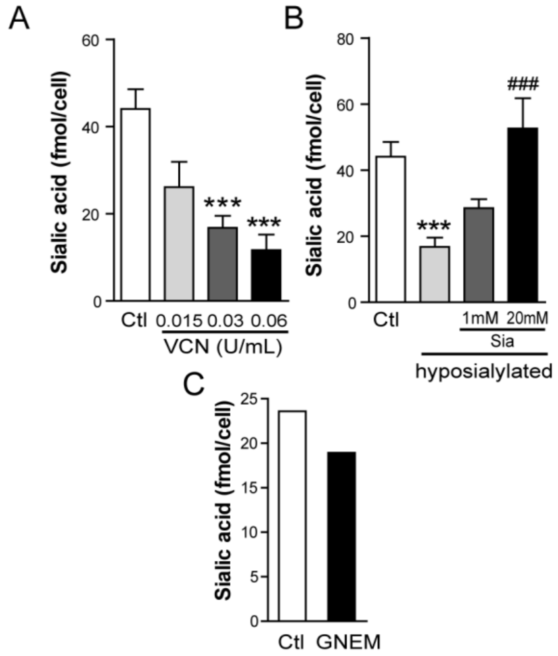

Supplementary Figure S2. Quantification of sialic acid in VCN treated myotubes and GNE myopathy myoblasts. (A) Sialic acid concentration measured by the resorcinol periodate method in C2C12 myotubes treated for 24 h with 0.015, 0.03 or 0.06 U/mL VCN. Data are the mean ± SEM of n = 3-9 independent experiments. ***p<0.001 *vs* control. **(B)** Sialic acid concentration in C2C12 myotubes treated with 0.03 U/mL VCN for 24 h (hyposialylated) and hyposialylated myotubes further treated with 1 mM or 20 mM Neu5Ac (Sia) for additional 24 h. Data are the mean ± SEM of n = 3-9 independent experiments. ***p<0.001 *vs* control, $^{###}$p<0.001 *vs.* hyposialylated. **(C)** Sialic acid concentration in control (Ctl) and GNE myopathy myoblasts (GNEM). Data comes from one independent experiment.

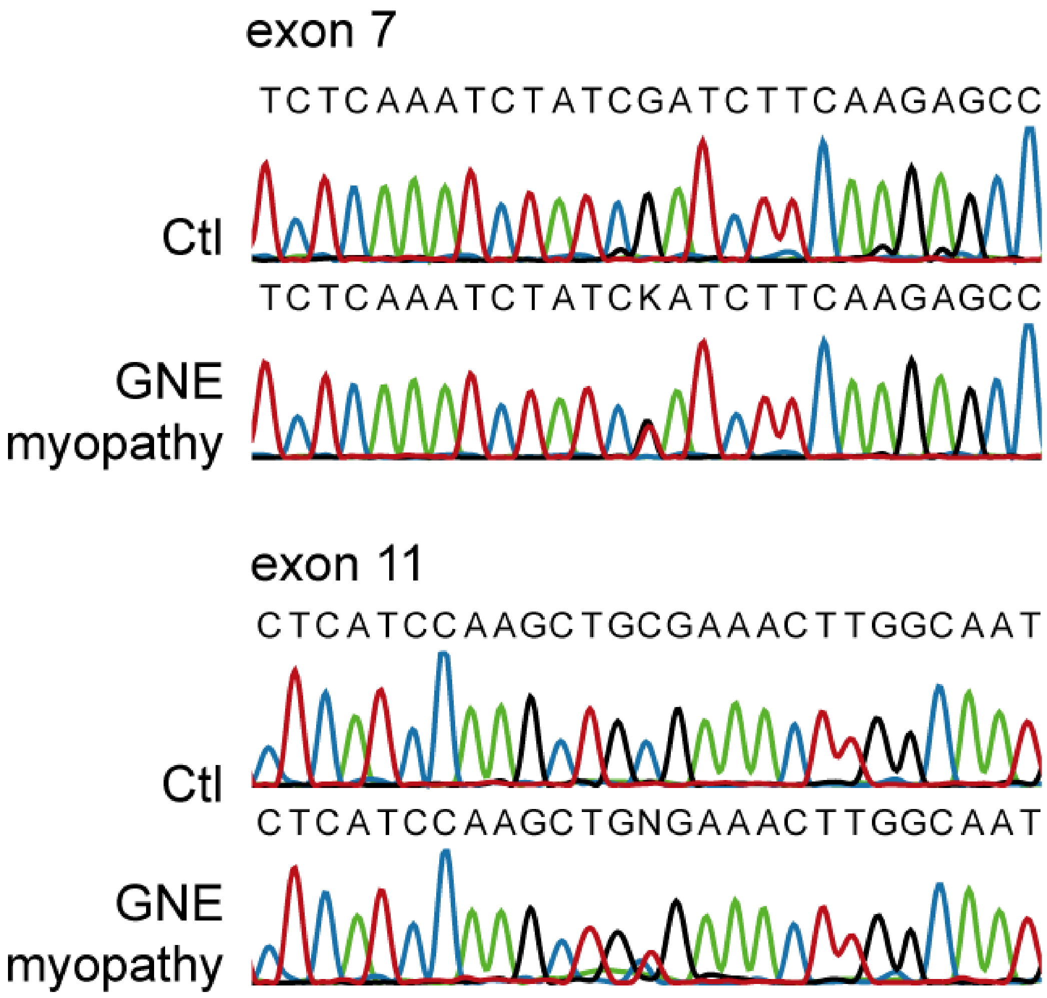

Supplementary Figure S3. *GNE* mutations in GNE myopathy myoblasts. Sequencing analysis of the exons 7 and 11 of *GNE* gene in myoblasts from a control donor and a patient with GNE myopathy showing a mutation in exon 7 (p.D378Y c.1132G>T) and a mutation in exon 11 (p.A631V c.1892C>T).

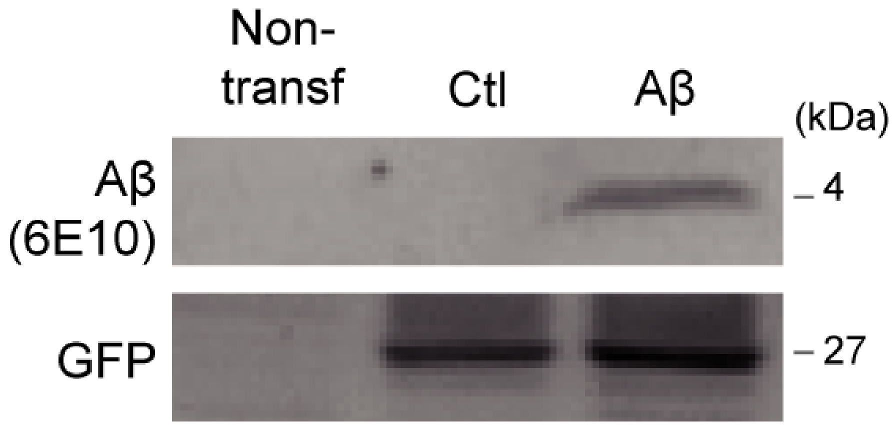

Supplementary Figure S4. Aβ expression in transfected myotubes. Western Blot analysis of Aβ and GFP expression in C2C12 myotubes without being transfected, transfected with GFP (Ctl) or transfected with AB-IRES-GFP (Aβ).

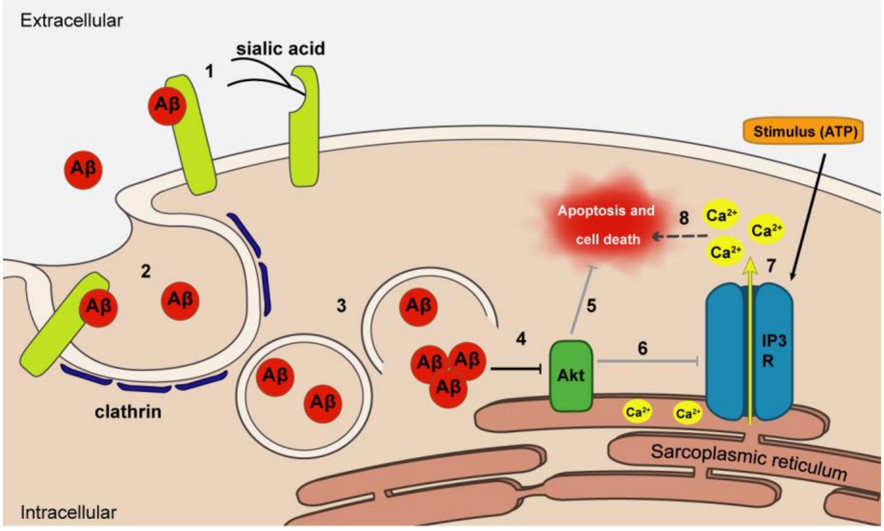

Supplementary Figure S5. Proposed model for internalization of Aβ leading to apoptosis in GNE myopathy. In control conditions, sialic acid would be interfering with Aβ endocytosis. However, when cell membrane is hyposialylated (1), Aβ may have access to receptors or binding molecules allowing its internalization, which is clathrin mediated (2). Increased Aβ endocytosis would raise intracellular levels of aggregated Aβ (3). Aβ oligomers would impair Akt activation (4) which could lead to apoptosis induction (5). Furthermore, IP_3R would be less restrained by Akt (6) inducing an increase in Ca^{2+} release from the ER to the cytosol (7), further aggravating apoptosis and cell death (8).
